# Preconditioning by aerobic exercise reduces acute ischemic renal injury in rats

**DOI:** 10.14814/phy2.14176

**Published:** 2019-07-19

**Authors:** Weslei V. de Lima, Iria Visona, Nestor Schor, Waldemar S. Almeida

**Affiliations:** ^1^ Nephrology Division, Department of Medicine Federal University of São Paulo (UNIFESP‐EPM) São Paulo SP Brasil; ^2^ Pathology Department Federal University of São Paulo (UNIFESP‐EPM), São Paulo SP Brasil

**Keywords:** Activated caspase‐3, acute kidney injury, apoptosis, autophagy, exercise preconditioning

## Abstract

Acute kidney injury (AKI) can be defined as the sudden loss of renal function associated with structural changes in the kidneys. Currently, 13.3 million people die of AKI around the world. Normally aerobic exercise is used both as/for the treatment and prevention of high blood pressure, metabolic disease and Diabetes mellitus (DM). Nevertheless, exercise preconditioning must be a crucial resource in the prevention and mitigation of AKI. The aim of this study was to evaluate the effects of the exercise preconditioning on renal IR (ischemic/reperfusion) experimental model. Male Wistars rats were divided into three groups (*n* = 9): sham (S), ischemic/reperfusion (IR), exercise + ischemic/reperfusion (EX + IR). IR renal injury was induced by clamping the bilateral renal artery for 45 min. The rats were subjected to exercise 5 days a week for 4 weeks with progressive intensity and duration. The group treated with exercise preconditioning, showed additional improvements in various parameters, including serum creatinine, proteinuria, and decrease of the severity of the tubular injury and activated caspase‐3 levels (*P* < 0.05). The previous aerobic exercise‐induced renoprotection in the IR injury. We anticipate that the practice of physical exercise in healthy individuals can also be useful for the prevention and attenuation of AKI.

## Introduction

Acute kidney injury (AKI) can be defined as the sudden loss of renal function associated with structural changes in the kidneys (Lameire et al. 2005; Endre et al. 2016). Currently, 13.3 million people die of AKI around the world (Nash et al. 2002; Ronco et al. 2010). Normally, aerobic exercise is used both as/for the treatment and prevention of high blood pressure, metabolic disease and DM (Colberg et al. 2010; Garber et al. 2011; Araujo et al. 2012). Nevertheless, exercise preconditioning must be an important resource in the prevention and mitigation of AKI.

Tissue damage of a particular organ due to ischemia is intensified in the moment of reoxygenation, during reperfusion. This is a process that is considered more harmful than ischemia itself. This mechanism of tissue injury is called reperfusion injury or ischemia‐reperfusion (IR) injury (Lee et al. 2013). During reperfusion, the blood flow restoration is often associated with an intensified tissue injury and intense inflammatory response (Pin‐Barre and Laurin, 2015). Reperfusion damage is identified specifically with the development of receptive oxygen species (ROS), endothelial cell damage, expanded vascular permeability, and the activation of platelets, neutrophils, and cytokines (Subramaniam et al. 2015). Renal IR may be shown histomorphology and biochemically. Both inflammation and apoptosis coexist in renal IR injury. During hypoxia, caspase activity increases as a result of intracellular Ca^2+^ accumulation. Caspase becomes activated in ischemic tissues and is an indicator of cell death (Thompson et al. 2007; Subramaniam et al. 2015). These changes, which can be observed in tubular cells, may cause the loss of brush borders of proximal tubular cells and spill out from the basement membrane of the cells into the tubular lumen, with eventual tubular obstruction (Kroshian et al. 1994; Rajasekaran et al. 2003).

The kidney is a vital organ in which preconditioning can probably produce a protective effect, given the need for high energy and a complex vascular network. Therefore, practical solutions must be found to not only prevent the development of the kidney lesion but also reduce the subsequent renal disorders. Previous studies have shown the beneficial effects of exercise on brain damage caused by ischemia and reperfusion in animal models of cerebral ischemia (Osada et al. 1999). However, the protective effects of exercise training from ischemia and reperfusion have still poorly understood. This study aimed to investigate the effects of exercise preconditioning on renal function following the IR in male rats.

## Methods

### Animals

Adult male Wistars rats (250–280 g) were bred in the CEDEME animal facility (UNIFESP, Brazil) and maintained in a temperature‐controlled room (23 ± 1°C) with a 12:12 h light–dark cycle (lights on at 7 am) with free access to food and water in standard polypropylene cages. All experiments were performed after approval of Institutional animal ethical committees, and the experimental protocol was approved by the Ethical Committee of UNIFESP (#826361).

### Experimental protocol

All rats underwent baseline urine and blood samples and then underwent IR. The animals were divided into three groups (*n* = 9): sham (S), ischemic/reperfusion (IR), exercise + ischemic/reperfusion (EX + IR). At the end of the experiments, the rats were sacrificed, and blood, urine and tissue samples were collected.

### Preconditioning aerobic exercise

The trained animals were adapted for 5 days before the beginning of the physical training. Physical training was performed between 40–60% of the exercise stress test in an adapted treadmill. The training occurred five times a week for 4 weeks with progressive intensity and duration as previously described by literature (Guimarães et al. 2010; Liu et al. 2012; Nakagawa et al. 2012)). A new exercise test was performed every 2 weeks to adjust the intensity of the physical training.

### Induction of AKI

IR renal injury was induced by clamping the bilateral renal artery for 45 min. The animals were anesthetized with ketamine (90 mg/kg) and xylazine (10 mg/kg) via intramuscular injections using strict hemostasis and aseptic techniques. After the preparation of the skin, the mouse was placed on the blankets of a homeothermic module and covered with sterile gauze. The animal was immobilized on a heated surgical table. After the end of the period, the clips were removed and observed until complete reperfusion, and no traumas in the kidneys. The animals were maintained on heating by indirect lighting until the complete recovery of the anesthesia and 48 h after the surgery.

### Biochemical study

Urinary protein, blood urea nitrogen (BUN), and plasma creatinine were evaluated with standard kits (Lab Test Diagnostics, Brazil) using an automated colorimetric technique. In addition, baseline measurements of body weight and urine volume were performed.

### Histopathology

The kidneys were fixed in 10% neutral buffered formalin. Paraffin‐embedded sections (3 *µ*m thick) were cut and stained with hematoxylin and eosin (HE) and Periodic Acid–Schiff's reaction. Using an optical microscope, a qualified pathologist performed all morphologic evaluations, being unaware of the nature of the treatment applied. The whole histological sections of each group were examined, in order to search for histological alterations in the tubules, both cortical and medullary. The degree of injury was measured by a semi‐quantitative analysis, as follows: Grade 0 – minimum (less than 10%); Grade 1 – mild (between 11 and 25%); Grade 2 – moderate (between 26 and 50%); Grade 3 – severe (between 56 and 75%); Grade 4 – very severe (over 76%).

### Immunohistochemical staining

For Immunohistochemical analysis, kidney sections were deparaffinized and rehydrated, and to expose the antigens, they were boiled in a target retrieval solution (TRIS buffer pH 9.0) for 30 min. Endogenous peroxidase activity was blocked with 3% H_2_O_2 _for 15 min at room temperature. Nonspecific binding was prevented by incubating the sections with a protein blocker (Dako, CA). Sections were incubated overnight at 4°C with primary rabbit anti‐caspase‐3 active monoclonal antibody (1:100, Santa Cruz Biotechnology, CA). After washing with TBS, the sections were incubated with horseradish peroxidase‐conjugated polymer (Dako) for 30 min at room temperature. Slides were rinsed with PBS, and the antibody–antigen reactions were visualized with 3, 3’‐diaminobenzidine (Dako, CA). The sections were lightly counterstained with hematoxylin. Analyses were performed using light microscopy (Leica Imaging Systems) at 20× magnification, and the stained proteins were quantified using Corel Photo‐Paint 12 and UTHSCSA – Image Tool software. The picture analysis was performed in two phases: first, measurement of the tissue area with the subtraction of the blank areas on the picture; second, calculating the percentage of staining present on the first phase picture.

### Statistical analysis

The results are presented as the mean ± standard deviation. Comparisons among different groups were evaluated using multiple analyses of variance (ANOVA) followed by Tukey's *post hoc* test. The tubulointerstitial fibrosis index was analyzed using the Kruskal–Wallis test with Bonferroni correction. The level of statistical significance was defined as *P* < 0.05.

## Results

### Body weight

**Figure 1:**
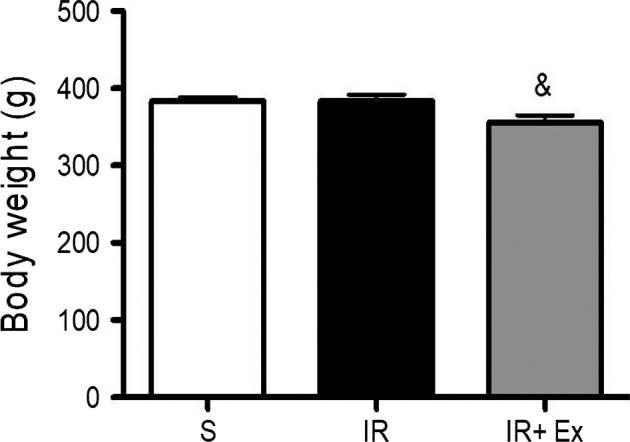
The effects of exercise preconditioning on body weight in IR rats. Values are expressed as the means ± SD. * (*P* < 0.05) versus S, # (*P* < 0.05) versus IR, & (*P* < 0.05) versus EX + IR.

All of the animals showed an increase in final body weight (Table 1 and Fig. 1). Exercise preconditioning IR animals showed body weight reduction when compared with sham and IR animals (sham 383 ± 16; EX + IR 356 ± 26 and IR 384 ± 24, *P* < 0.05).

**Table 1:** Baseline and final body weight parameters

Groups	Initial	Final	n
S	301 ± 1.9	383 ± 16^*^	9
IR	301 ± 1,9	384 ± 24^*^	9
IR + EX	302 ± 3,5	356 ± 26^*†^	9

Values are expressed as means ± SD. * (*P* = <0.05) versus initial S, IR and EX + IR. ^†^ (*P* = <0.005) versus final S and IR.

### Measurement of renal function

#### Blood urea nitrogen (BUN) and plasma creatinine

**Figure 2:**
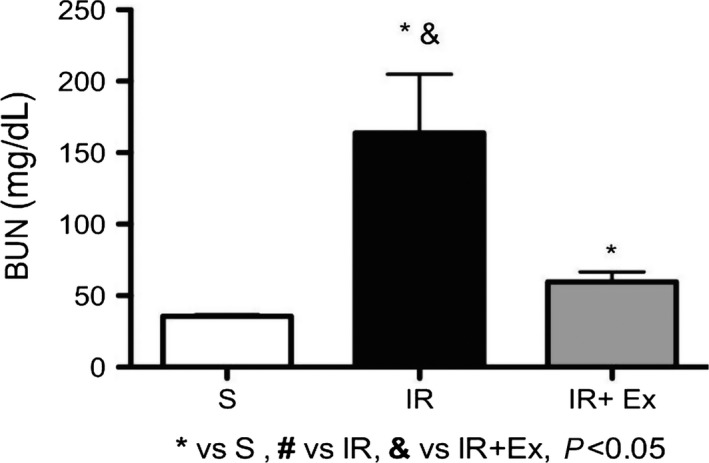
The effects of exercise preconditioning on blood urea nitrogen in IR rats. Values are expressed as the means ± SD. & (*P* < 0.05) versus EX + IR. * (*P* < 0.05) versus S, # (*P* < 0.05) versus IR, & (*P* < 0.05) versus EX + IR.

**Figure 3:**
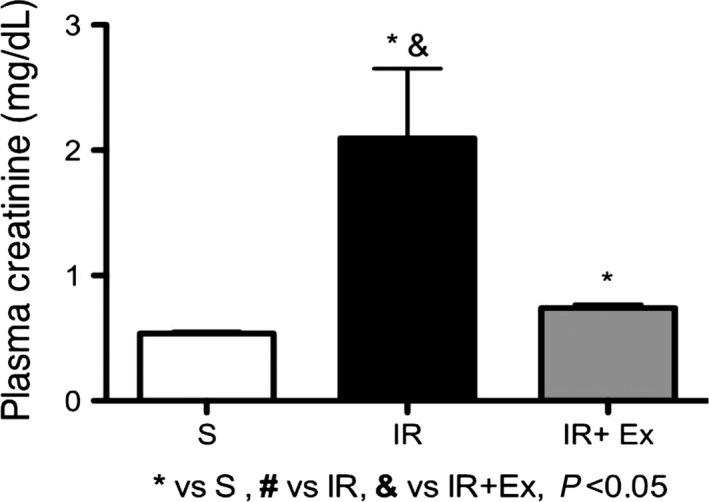
The effects of exercise preconditioning on plasma creatinine (mg/dL) in IR rats. Values are expressed as the means ± SD. * (*P* < 0.05) versus S, # (*P* < 0.05) versus IR, & (*P* < 0.05) versus EX + IR.

As shown in Figures 2 and 3, untreated animals presented higher values of serum creatinine and urea compared sham and treated group (*P* < 0.05). Notably, exercise preconditioning IR treatment significantly prevented the increases in plasma creatinine and BUN levels and improved the renal function compared to the IR group (*P* < 0.05).

### Proteinuria

**Figure 4:**
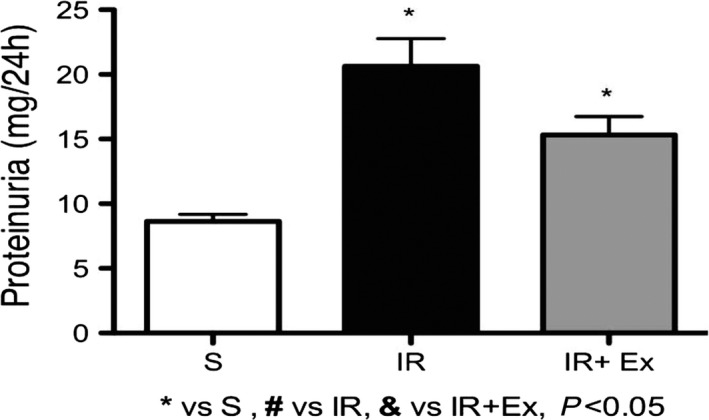
The effects of exercise preconditioning on proteinuria in IR rats. The progression of proteinuria is significantly inhibited by exercise preconditioning. Values are expressed as means ± SD. * (*P* < 0.05) versus S, # (*P* < 0.05) versus IR, & (*P* < 0.05) versus EX + IR.

Exercise preconditioning IR treatment significantly prevented the increase in proteinuria levels compared to the IR group (EX + IR = 15 ± 4, IR = 20 ± 6, *P* < 0.05) (Fig. 4). Furthermore, the animals of the sham group presented values of excretion of proteins in the urine similar to the initial parameters.

### Histopathology

**Figure 5:**
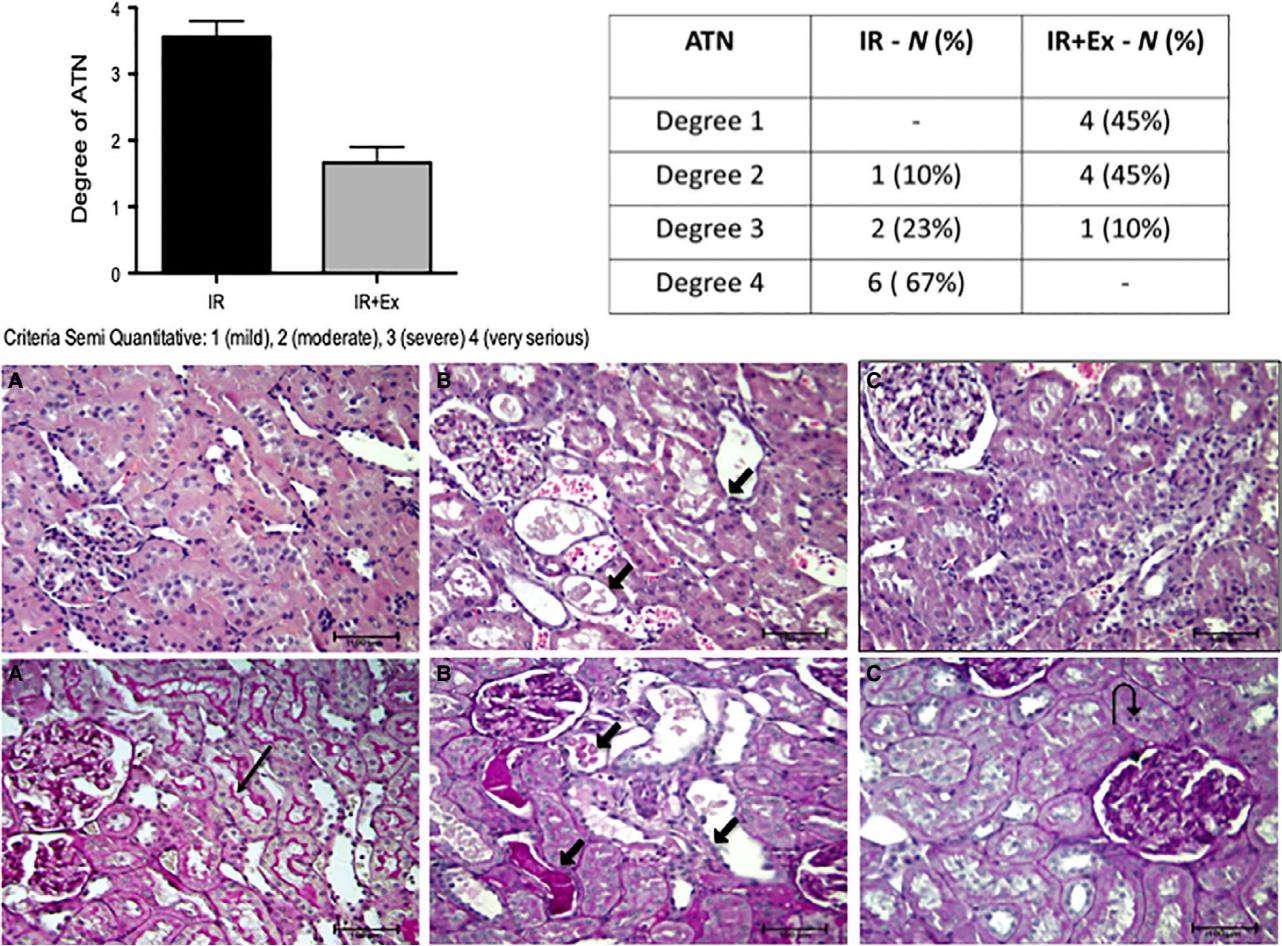
Microscopic aspects of renal sections with the main injuries observed in each group. (A) Sham: Normal renal structure, with preserved tubular brush border (long black arrow). (B) IR group. Acute tubular injury showing cell lining denudation, dilated tubules with cast material and cytoplasmic debris in tubular lumen, best seen here in the PAS stain (short black arrow). The glomerulus appears unremarkable. (C) EX + IR group. We observe less tubular injury, with only focal reduction of the brush border and regeneration of the cells that seem more crowded, eventually having one mitotic figure (curve black arrow). [HE (top) and PAS (bottom); ×200]. Graph and table show the values expressed as the means ± SD. * (*P* < 0.05) versus sham, # (*P* < 0.05) versus IR, & (*P* < 0.05) versus EX + IR.

The main affected renal structures were the tubules, specially the proximal ones, showing degenerative and necrotic alterations, such as loss of brush border, denudation of the tubular cell lining and dead cells occupying the tubular lumen. Those are characteristics of ischemic injuries. In the IR group, 67% of the animals had degree 4, that is, very severe tubular injuries. On the other hand, in the EX + IR group, 90% of the animals had grades 1 and 2 (mild to moderate injuries). Comparing them, the group EX + IR had less tubular injuries, reduction of tubular cells degenerative changes and signs of cell regeneration. In the Figure 5, we see the most representative morphological features of each group.

### The effects of exercise preconditioning IR on apoptosis cell death

**Figure 6:**
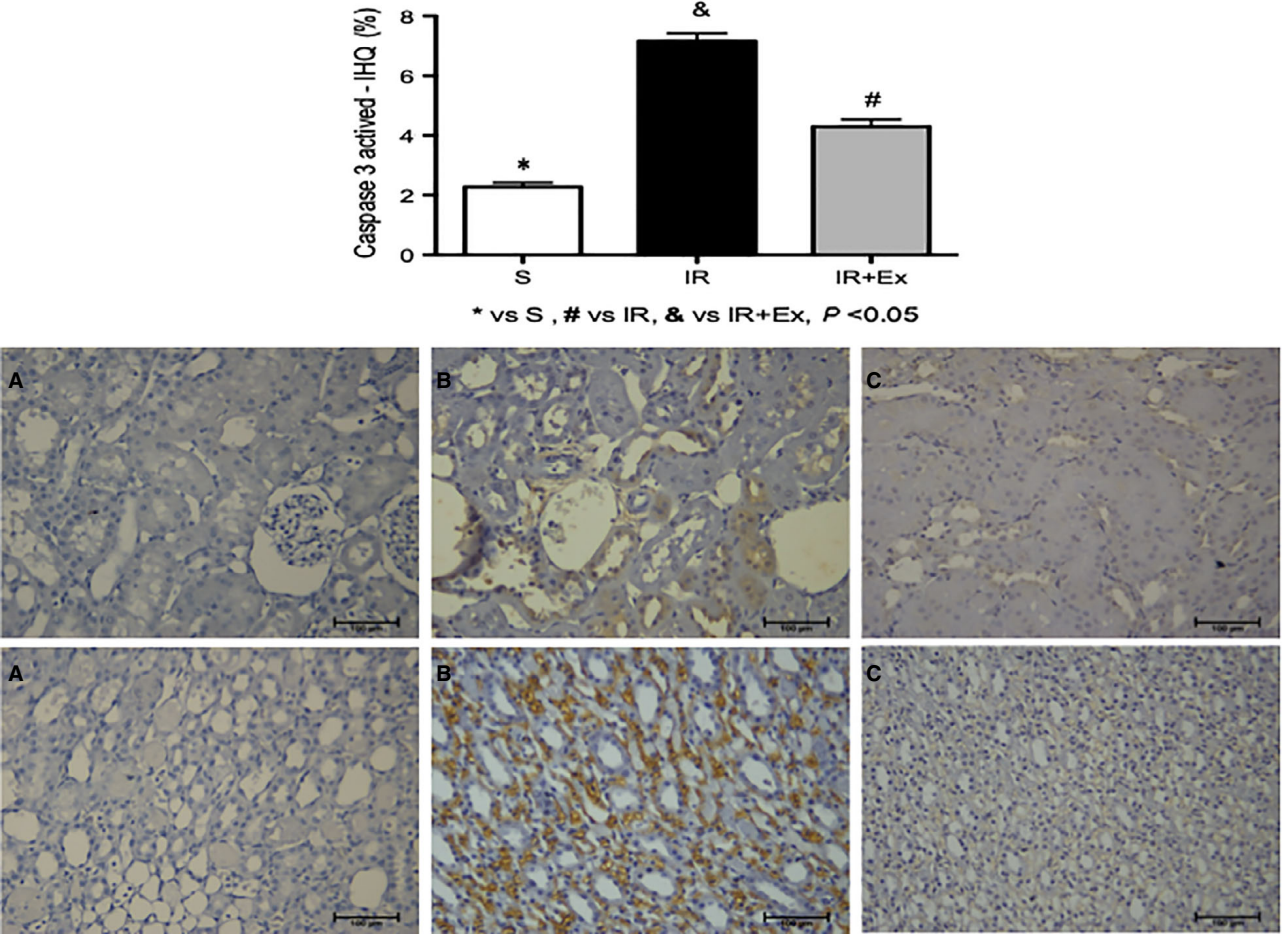
Caspase activation in tubular cells during renal ischemia‐reperfusion injury in sedentary and exercised rats. Male rats sedentary and exercised were subjected to 45 min of renal ischemia followed by 48 h of reperfusion. Renal tissues were fixed for immunofluorescence using an antibody specific for active caspase‐3. (A) Representative images of light marking for caspase‐3 active in renal tubular cells of the sham group. (B) Representative images of less intense marking for caspase‐3 active in renal tubular cells of the EX + IR group. (C) representative images of intense marking for caspase‐3 active in renal tubular cells of the IR group. Values are expressed as means ± SD. * (*P* < 0.05) versus sham, # (*P* < 0.05) versus IR, & (*P* < 0.05) versus EX + IR. [cortex (top) and medulla (bottom); 200×].

The expression of activated caspase‐3 in renal tissue was higher in all IR animals compared to sham rats (Fig. 6). Furthermore, many apoptotic cells were observed in proximal and medullary tubular cells of group IR compared to EX + IR group, that showed less increase in renal caspase‐3 expression (*P* < 0.05).

## Discussion

We have demonstrated that regular, progressive aerobic exercise performed prior to IR acute renal injury was able to attenuate plasma creatinine and urea levels, decrease the severity of the tubular injury, and active caspase‐3 levels 48 h after reperfusion, in rats Wistars. The damage of renal morphology, as the presence of interstitial edema, mononuclear infiltrates, loss of brush border and tubule epithelial cells were of lower severity in the group that was previously submitted to regular aerobic exercise, compatible with the improved renal function. The kidney is very sensitive to hypoxia and vulnerable to ischemic or hypoxic injury because of the renal vascular anatomy and the high energy consumption of renal tubular epithelial cells (Kroshian et al. 1994; Haase, 2013; Linkermann et al. 2014). The mechanism of renal I/R injury is extremely complex, however, much of the damage is mediated by reactive oxygen species (ROS) during reperfusion (Tesch, 2010). ROS activate inflammatory cells resulting in the release of interleukin (IL), tissue necrosis factor, and other inflammatory factors, promoting cell apoptosis and increasing tissue damage during reperfusion. In this study, we still show that active caspase‐3 levels were significantly lower in the previously exercised AKI group. When activated, the caspases are able to cleave several key autophagy proteins that result in suppression of autophagy. The cleaved fragments of autophagy proteins produced have been shown to have a proapoptotic function (Zhu et al. 2010; Oral et al. 2016; Tsapras and Nezis, 2017). Activation of autophagy can be stimulated in several ways, including infection, caloric restriction, and exercise. In addition to cell metabolism and the cell survival/death mechanism, autophagy plays an important role in maintaining cellular homeostasis in skeletal muscle, especially during exercise where energy demand can be extremely high. By degrading macromolecules and subcellular organelles through the fusion of autophagosomes and lysosomes, materials useful as amino acids can be released and reused to support normal cell metabolism (Kaushal and Shah, 2016). Recent studies demonstrate that basal autophagy in the kidney is vital for the normal homeostasis of the proximal tubules (Jiang et al. 2010; Kimura et al. 2011). Autophagy deletion in proximal tubules worsened the tubular injury and renal function (Liu et al. 2012; Kaushal and Shah, 2016). Both autophagy and apoptosis are generally into the common stimulus. The regulation of shared pathways between autophagy and apoptosis determines the outcome of cell fate for either cell survival or cells death (Pattingre et al. 2005). Thus, we speculated that the reduction of activated caspase‐3 in the group of previously exercised animals may have contributed to greater autophagy and protection of renal tubular damage. In the recent past (Lee et al. 2013), it was demonstrated that exercise preconditioning reduces acute ischemic renal injury in HSP70.1 knockout mouse.

The effects of exercise have been shown to be an autophagic inducer, cellular autophagic responses to exercise in skeletal muscle appear to be varied in different exercise protocols and disease models (Lenhare et al. 2017; Jeong et al. 2017). Exercise is the greatest physiological stress that our bodies experience. Given the physiological stress associated with exercise and the adaptations that occur to handle this stress, it is not surprising that exercise training is known to prevent or effectively treat a multitude of degenerative conditions including cardiovascular disease, cancer, diabetes, depression, Alzheimer's disease, Parkinson's disease, and many others (Guimarães et al. 2010; Pin‐Barre and Laurin, 2015). Many of the health benefits of exercise are mediated by the mammalian/mechanistic target of rapamycin (mTOR), not only within the working muscle but also in distant tissues that are susceptible to IR (Nakagawa et al. 2012; Kaushal and Shah, 2016). In view of this, we suggest that regular and previously applied aerobic physical training induced an initial and or more intense autophagy to the detriment of the lower level of activated caspase‐3 resulted in a lower degree of apoptosis of the tubular cells, according to our biochemical and morphological characteristics, presented in a renal model of animal I/R.

We have not identified the mechanism by which exercise improves tolerance to ischemia and attenuates the tissue renal damage caused by ischemia‐reperfusion injury. However, moderate aerobic exercise decreases the development of cardiovascular diseases such as hypertension, helps control cholesterol and weight, preserves bone mass, improves glucose, oxygen and nutrient uptake into skeletal muscle, increases respiratory capacity and ATP (Guimarães et al. 2010; Zhu et al. 2010; Tsapras and Nezis, 2017). We suggest the better physical conditioning of trained animals may lead to better hemodynamic and metabolic conditions, and finally, cellular and molecular survival mechanisms of renal tissue. There is a growing interest in studying the role and regulation of the autophagy pathway because of the indication that autophagy is cytoprotective in response to various stresses. Recent studies have recognized that autophagy is selective in degrading specific targets including damage proteins and organelles. However, more studies are required to understand precise mechanisms of autophagy including mitophagy in AKI, and about 15–20% of patients with AKI progress to chronic kidney disease stage IV (Kaushal and Shah, 2016).

In conclusion, our results demonstrate that the amount of aerobic exercise previously applied was efficient to protect acute ischemic renal injury in rats. We suggest that previous aerobic exercise induced a higher degree of survival (autophagy) in relation to cellular death (apoptosis) due to the reduction of the activated caspase‐3 level. We anticipate that the practice of physical exercise in healthy individuals can also be useful for the prevention and attenuation of AKI.

## Conflict of Interest

None declared.
